# Supplementary material for: Anisotropic surface potentials induced by competitive ion adsorption enable the synthesis of branched cubic Pt mesocrystals
Source: Nat Commun. 2025 Nov 5;16:9758. doi: 10.1038/s41467-025-64494-9 (PMC12589459; doi:10.1038/s41467-025-64494-9)
Supplement: Supplementary file 1 — Supplementary Information [file 41467_2025_64494_MOESM1_ESM.pdf]

Supplementary Information for

Anisotropic surface potentials induced by competitive ion adsorption enable the synthesis of branched cubic Pt mesocrystals

Yuna Bae[1], Eun Mi Kim[2], Jaehun Chun[1], Zihua Zhu[3], Trevor H. Moser[3], Hanlei Zhang[1], Jaeyoung Heo[1], Yun Kyung Shin[4], Hua Zhou[5], James E. Evans[3], Emil C. S. Jensen[6], Kristian S. Mølhave[7], Kristen A. Fichthorn[2]*, James J. De Yoreo[1,8]*, Dongsheng Li[1]*

*Corresponding author. Email: fichthorn@psu.edu (K.A.F.); James.DeYoreo@pnnl.gov (J.J.D.Y.); Dongsheng.Li2@pnnl.gov (D.L.)

Table of Contents	Page
Supplementary Note 1	2–5
Supplementary Note 2	6–7
Supplementary Figures 1–26	8–34
Supplementary Tables 1–3	35–37
Supplementary references	38–39

Supplementary Note 1. Probabilistic interpretation of the balance between rotational Brownian motion and electrostatic torque for alignment of nanocrystals by anisotropic electrostatic interactions

Note that all interactions were calculated with nanoparticles with a diameter of 3 nm.

To describe the consequences of anisotropic electrostatic interactions, we use an orientational probability distribution analogous to that employed by Doi and Edwards[1]. This leads to a Smoluchowski equation describing the orientation of one crystallite relative to another in terms of a director ($\mathbf{p}$) lying along a crystallographic axis, a unit vector denoting the orientation (e.g. a unit vector perpendicular to (111) or (100) surfaces in this case). The orientational probability distribution function (Ψ) can be expressed as,

$$\frac{\partial \Psi}{\partial t} = \mathrm{D_r}\left(\mathbf{p} \times \frac{\partial}{\partial \mathbf{p}}\right) \cdot \left[\mathbf{p} \times \frac{\partial \psi}{\partial \mathbf{p}} + \frac{\psi}{\mathrm{k_B}T}\mathbf{p} \times \frac{\partial U}{\partial \mathbf{p}}\right]. \quad \text{(S1)}$$

This depends on the orientation vector ($\mathbf{p}$) (a unit vector perpendicular to (111) or (100) surfaces in this case), the center-to-center distance (D), and time. $\mathrm{D_r}$ is the rotational diffusivity of a crystallite, $\mathrm{k_B}$ is the Boltzmann constant, and T is the absolute temperature. The probability distribution for a particular orientation arises from the anisotropic electrostatic interaction potential (U) at equilibrium can be expressed as,

$$\frac{\partial \ln \Psi}{\partial \mathbf{p}} = -\frac{1}{\mathrm{k_B}T}\frac{\partial U}{\partial \mathbf{p}}, \quad \text{(S2)}$$

indicating that the orientation distribution function embodies a balance between electrostatic ($\partial U/\partial \mathbf{p}$) and Brownian ($\mathrm{k_B}T$) contributions. It follows that the orientation of one crystallite relative to the other is described by

$$\Psi = \tilde{C}\exp\left(-\frac{1}{\mathrm{k_B}T}\int \mathrm{d}p_i\, \Gamma_i\right), \quad \text{(S3)}$$

where $\tilde{C}$ is an integration constant and Γ_i represents the torque *(i=x,y,z)* as determined from the anisotropic electrostatic interaction.

To describe the facet-dependent alignment, a simple 2D-based model with two truncated octahedra, where the rotational motion of the director of crystallite-2 is confined to the *xy*-plane (Supplementary Fig. 24). The normal vector ($\mathbf{p}$) of the facet of crystallite-1 points along the *y*-coordinate while that of crystallite-2 is tilted at an angle (θ_B) with respect to the *y*-coordinate. Crystallite-1 is fixed as the reference body and crystallite-2 undergoes rotational motion due to Brownian motion and the anisotropic electrostatic interaction. In the *xy* plane, the alignment between the crystallites is described by the *x* component of $\mathbf{p}$. $p_x = 0$ corresponds to $\{100\}-\{111\}$ alignment and $p_x = 1/\sqrt{2}$ corresponds to $\{100\}-\{100\}$ alignment. The probability density function for orientation in the *xy* plane is

$$f(p_x) = \frac{\exp\left(-\frac{1}{\mathrm{k_B}T}\int \mathrm{d}p_x\, \Gamma_x\right)}{\sqrt{2}\int_0^{\frac{1}{\sqrt{2}}} \mathrm{d}\tilde{p}_x \exp\left(-\frac{1}{\mathrm{k_B}T}\int \mathrm{d}p_x\, \Gamma_x\right)}, \quad \text{(S4)}$$

From the probability as a function of orientation, we can determine the degree of preferential alignment using a ratio of probabilities.

The electrostatic interaction energy (U_{elec}) between two spherical particles were calculated by the following equation[2],

$$U_{\mathrm{elec,100}} = \pi\varepsilon_0\varepsilon_\mathrm{r}\frac{r_1 r_2}{D}\left(\frac{\mathrm{k_B}T}{ze}\right)^2 [(y_1 + y_2)^2 \ln(1 + e^{-\kappa h}) + (y_1 - y_2)^2 \ln(1 - e^{-\kappa h})], \quad \text{(S5)}$$

where y is a normalized surface potential (=$e\psi_\mathrm{s}/\mathrm{k_B}T$), ψ_s is a dimensional surface potential, κ^{-1} is the Debye length of the solution, ε_0 is the permittivity of free space, ε_r is the relative permittivity of the solvent, e is the charge of the electron, z is the valence of ions, D is the center-to-center distance between two NPs, h is a minimum separation between two NPs, and r_1 and r_2 is the radius of two NPs. This equation was proven to be valid for $\kappa r \approx 1$ or less[2]. In our system, $\kappa r \approx 0.43$ and y <2.45.

The Debye length κ^{-1} was calculated using

$$\kappa^{-1} = \left(\frac{\varepsilon_0\varepsilon_\mathrm{r}\mathrm{k_B}T}{\sum_i 1000\mathrm{N_A}C_i e^2 z_i^2}\right)^{\frac{1}{2}} \approx 3.46\ \mathrm{nm} \quad \text{(S6)}$$

where $\mathrm{N_A}$ is Avogadro's number and C is the concentration of ions (mol $\mathrm{L^{-1}}$).

To avoid the underestimation of electrostatic interactions between NPs, ψ_s can be corrected by the following equation[3],

$$\psi_\mathrm{s} = \xi\left(1 + \frac{1}{\kappa r}\right)\hat{\mathrm{e}}, \quad \text{(S7)}$$

where ξ is the measured zeta potential, r is the radius of the NPs, and ê is Euler's number approximately equal to 2.71828.

We set the variables as follows for simplicity.

$$a = \psi_\mathrm{s,100};\ b = \psi_\mathrm{s,111};\ c = \ln(1 + e^{-\kappa h});\ d = \ln(1 - e^{-\kappa h});\ A = \pi\varepsilon_0\varepsilon_\mathrm{r}\frac{r_1 r_2}{D};\ p_x = \sin\theta_\mathrm{B}$$

Every π/2-angle rotation will become identical. To compare the (100)–(100) alignment with the (100)–(111) alignment, we can only consider angles within the range $0 < \theta_\mathrm{B} < \pi/4$ and build a simple model for angular orientation nature associated with electrostatic interactions. The surface potential of crystallite-2 can be expressed via θ_B with respect to the y-coordinate.

$$\begin{aligned}\psi_\mathrm{s,2} &= \psi_\mathrm{s,111}\cos 2\theta_\mathrm{B} + \psi_\mathrm{s,100}\sin 2\theta_\mathrm{B} = \psi_\mathrm{s,111}(1 - 2\sin^2\theta_\mathrm{B}) + \psi_\mathrm{s,100} 2\sin\theta_\mathrm{B}\cos\theta_\mathrm{B} \quad \text{(S8)}\\ &= b(1 - 2p_x^2) + 2ap_x\sqrt{1 - p_x^2}\end{aligned}$$

The electrostatic interaction energy (U_{elec}) between two spherical particles and the torque (Γ_x) in the x-coordinate are calculated by the following equations,

$$\begin{aligned}U_{\mathrm{elec,100}} &= \pi\varepsilon_0\varepsilon_\mathrm{r}\frac{r_1 r_2}{D}\left[\left(\psi_\mathrm{s,100} + \psi_\mathrm{s,2}\right)^2 \ln(1 + e^{-\kappa h}) + \left(\psi_\mathrm{s,100} - \psi_\mathrm{s,2}\right)^2 \ln(1 - e^{-\kappa h})\right] \quad \text{(S9)}\\ &= A\left[\left(a\left(1 + 2p_x\sqrt{1 - p_x^2}\right) + b(1 - 2p_x^2)\right)^2 c + \left(a\left(1 - 2p_x\sqrt{1 - p_x^2}\right) - b(1 - 2p_x^2)\right)^2 d\right]\end{aligned}$$

$$\Gamma_{x,100} = \frac{\partial U_{\mathrm{elec,100}}}{\partial p_x} \quad \text{(S10)}$$

$$= 4A\left\{a\left(\sqrt{1-p_x^2} - \frac{p_x^2}{\sqrt{1-p_x^2}}\right) - 2bp_x\right\}\left\{\left(b(1-2p_x^2) + 2ap_x\sqrt{1-p_x^2}\right)(c+d) + a(c-d)\right\}$$

To compare the (111)−(111) alignment with the (111)−(100) alignment, we can apply the simple model in the same way (Supplementary Fig. 25).

$$\psi_{\mathrm{s},2} = \psi_{\mathrm{s},100}\cos 2\theta_{\mathrm{B}} + \psi_{\mathrm{s},111}\sin 2\theta_{\mathrm{B}} = \psi_{\mathrm{s},100}(1 - 2\sin^2\theta_{\mathrm{B}}) + \psi_{\mathrm{s},111}2\sin\theta_{\mathrm{B}}\cos\theta_{\mathrm{B}} \quad \text{(S11)}$$
$$= a(1-2p_x^2) + 2bp_x\sqrt{1-p_x^2}$$

$$U_{\mathrm{elec},111} = \pi\varepsilon_0\varepsilon_{\mathrm{r}}\frac{r_1 r_2}{D}\left[\left(\psi_{\mathrm{s},111} + \psi_{\mathrm{s},2}\right)^2 \ln(1 + e^{-\kappa h}) + \left(\psi_{\mathrm{s},111} - \psi_{\mathrm{s},2}\right)^2 \ln(1 - e^{-\kappa h})\right] \quad \text{(S12)}$$
$$= A\left[\left(b\left(1 + 2p_x\sqrt{1-p_x^2}\right) + a(1-2p_x^2)\right)^2 c + \left(b\left(1 - 2p_x\sqrt{1-p_x^2}\right) - a(1-2p_x^2)\right)^2 d\right]$$

$$\Gamma_{x,111} = \frac{\partial U_{\mathrm{elec},111}}{\partial p_x} \quad \text{(S13)}$$
$$= 4A\left\{b\left(\sqrt{1-p_x^2} - \frac{p_x^2}{\sqrt{1-p_x^2}}\right) - 2ap_x\right\}\left\{\left(a(1-2p_x^2) + 2bp_x\sqrt{1-p_x^2}\right)(c+d) + b(c-d)\right\}$$

Contributions from neighboring crystallites are represented by the overall probability density function $f(p_x^1, p_x^2, \cdots, p_x^m)$ where m denotes the number of nearby crystallites.

$$f(p_x^1, p_x^2, \cdots, p_x^m) = [f(p_x)]^m = \frac{\exp\left(-\frac{m}{\mathrm{k_B}T}\int \mathrm{d}p_x\,\Gamma_x\right)}{\left[\sqrt{2}\int_0^{\frac{1}{\sqrt{2}}} \mathrm{d}\tilde{p}_x \exp\left(-\frac{1}{\mathrm{k_B}T}\int \mathrm{d}p_x\,\Gamma_x\right)\right]^m} \quad \text{(S14)}$$

The ratio of the probabilities for the {100}−{100} and {100}−{111} alignments (P_{100}) and the ratio of the probabilities for the {111}−{111} and {111}−{100} alignments (P_{111}) are,

$$P_{100} = \frac{f\left(p_x = \frac{1}{\sqrt{2}}\right)}{f(p_x = 0)} = \frac{C_1 \exp\left(-\frac{m_1 A}{\mathrm{k_B}T}\left((a-b)\left(b(c+d) + a(3c-d)\right)\right)\right)}{C_1 \exp\left(-\frac{m_1 A}{\mathrm{k_B}T} \times 0\right)} \quad \text{(S15)}$$

$$P_{111} = \frac{f\left(p_x = \frac{1}{\sqrt{2}}\right)}{f(p_x = 0)} = \frac{C_2 \exp\left(-\frac{m_2 A}{\mathrm{k_B}T}\left(-(a-b)\left(a(c+d) + b(3c-d)\right)\right)\right)}{C_2 \exp\left(-\frac{m_2 A}{\mathrm{k_B}T} \times 0\right)} \quad \text{(S16)}$$

Then, the ratio of the probabilities for the {100}−{100} and {111}−{111} alignments can be expressed as

$$\frac{P_{100}}{P_{111}} = \exp\left[-\frac{A}{(\mathrm{k_B}T)(a-b)\{(m_1 b + m_2 a)(c+d) + (m_1 a + m_2 b)(3c-d)\}}\right] \quad \text{(S17)}$$

Note that the normalization constant can be negligible by reduction of fraction. A truncated octahedron has six {100} facets and eight {111} facets, so in this case, m_1 and m_2 are 6 and 8,

respectively. Moreover, the torques required for both preferential alignments are greater than $k_B T$ when the separation is below around 2 nm (Supplementary Fig. 26).

In the early stage of the growth process, for $|\psi_{s,100}| < |\psi_{s,111}|$, P_{100}/P_{111} plotted as a function of separation shows that the preferential alignment along (100)−(100) increases significantly as separation decreases (Fig. 5d). This indicates that the torque experienced by particles is sufficient to align particles along the {100} facets, where the relative surface potential is lower. The zeta potentials at A_{tot}=30 m^2 L^{-1} in Fig. 5c were used in the calculations; ξ_{100} is −0.7 mV and ξ_{111} is −3.2 mV.

At the later stage of the growth process, for $|\psi_{s,100}| > |\psi_{s,111}|$, P_{111}/P_{100} is plotted as a function of separation shows that the preferential alignment along (111)−(111) increases significantly as separation decreases (Fig. 5e). The zeta potentials at A_{tot}=30 m^2 L^{-1} in Fig. 5c were used in the calculations; ξ_{100} is −7.5 mV and ξ_{111} is −4.5 mV.

Supplementary Note 2. Torque arising from dipole−dipole and van der Waals interactions

Each torque arising from dipole−dipole and van der Waals (vdW) interactions can be considered with contributions derived from the shape effect and the intrinsic material properties, respectively. We note that the primary NPs are a truncated octahedron (Supplementary Fig. 16).

Dipole-dipole interaction:

Although perfectly spherical and symmetric Pt NPs do not possess permanent dipole moments, structural or chemical asymmetries within the particles can result in nonuniform charge distributions that give rise to a net dipole moment. The truncated octahedron, however, is a highly symmetric structure, characterized in particular by inversion symmetry. In this geometry, each face has an identical counterpart on the opposite side of the particle, relative to its center. Since the dipole moment is a vector quantity that arises from asymmetric charge distributions, any dipole contributions generated by surface charges on individual facets cancel out due to this symmetry. Specifically, the dipole moments associated with each pair of square {100} facets and each pair of hexagonal {111} facets cancel one another. Even if the surface charge densities of the {111} and {100} facets differ, the overall distribution remains balanced because each type of facet is symmetrically arranged. As a result, the vector sum of dipole contributions from all hexagonal facets is 0, as is the sum from all square facets. Therefore, the net dipole moment of an ideal truncated octahedral Pt NP is 0.

Some materials, such as ZnO, have a permanent dipole moment due to their inherently asymmetrical internal crystal structure, whether spherical or symmetrical[4]. This intrinsic polarity creates dipole-dipole interactions with distinct orientations between particles, so the anisotropic torque generated by these interactions can be a crucial factor to consider alongside torques by other interparticle interactions.

vdW interaction:

Any nonspherical particle can experience a torque from vdW interactions due to its shape anisotropy[5,6]. The truncated octahedron belongs to the family of Archimedean polyhedra, in which all vertices lie on a common circumscribing sphere. Its overall geometry closely approximates a sphere so that the geometric transition between the faces and vertices of the truncated octahedron is much less pronounced compared to a distinct example such as cubic particles. This makes the variation in interaction energy for relative particle orientations insignificant. Thus, due to its high symmetry and near-sphericity, the vdW torque can be considered negligible when compared to that of more anisotropic shapes.

The Hamaker constant (A) is usually considered as a scalar property for a given material, derived from the frequency-dependent dielectric function of materials by Lifshitz theory[7]. In Lifshitz theory extended to anisotropic crystals, materials have different dielectric constants along different principal axes, meaning that the Hamaker constant becomes orientation-dependent[8]. However, in face-centered cubic (FCC) metals such as Pt, the dielectric response is highly isotropic due to the crystal's cubic symmetry. This isotropy implies an isotropic or nearly isotropic dielectric response. In other words, the polarizability (α) does not vary significantly with crystallographic orientation (i.e., {100}, {111}) or with the angle (θ) between interacting surfaces (i.e., relative particle orientation). To illustrate this aspect further, one can utilize the Hamaker's seminal work[9]; the Hamaker constant is proportional to the square of the polarizability. This can lead to a simple rationale that an angular dependence would be associated

with a polarizability difference in directionality on the specific facet. Thus, the Hamaker constant is unlikely to be orientation- and angular-dependent, resulting in negligible differences in the angular derivative ($\partial A(\theta)/\partial\theta$), and consequently in the vdW torque.

Importantly, both dipole–dipole torque and vdW torque originate from intrinsic material properties and therefore remain constant over time during growth process of branched cubic mesocrystals. This implies that they are not directly responsible for the switch in planes of attachment. Instead, the dominant factor is more likely the electrostatic torque resulting from ion competition, along with its directional switching. This case—that electrostatic torque dominates over other interactions like vdW and dipole-dipole torques—is not specific to the Pt NPs studied here but can be generalized to a wide range of materials, given that: 1) inherently polar crystals like ZnO are an exception rather than the rule, 2) many nanocrystals, particularly those synthesized via common methods, adopt either spherical or other simple geometric shapes with high symmetry, which minimizes shape anisotropy, and 3) the polarizability of adjacent facets is similar for any high-symmetry crystal class, resulting in a negligible orientation-dependent vdW torque. In contrast, the surface potential of adjacent facets will generally differ in magnitude and, often, in sign over significant pH ranges. For example, in a non-polar oxide like Fe_2O_3 (hematite), $\{100\}$, $\{012\}$, and $\{113\}$ facets exhibit different pH dependence of surface potential due to differences in atomic arrangements and termination on facets[10]. Similarly, in the cubic perovskite semiconductor $SrTiO_3$, the $\{100\}$ facets can be terminated with either SrO or TiO_2 layers, being intrinsically electrically neutral, while the $\{110\}$ facets are polar with two highly charged possible terminations, $SrTiO^{4+}$ and $(2O)^{4-}$, resulting in facet-dependent charging behavior upon varying pH[11]. Therefore, our study that correlates facet-dependent electrostatic interactions to orientational motions of nanocrystals provides important physical insights on OA for a wide range of materials. Furthermore, if the interplay among the three types of torque can be leveraged to control assembly orientation involving materials with anisotropic Hamaker constants, permanent dipole moments, or anisotropic shapes, it can be extended to a strategy for material synthesis via directed assembly for virtually any crystal class.

This last statement may seem to be inapplicable to cubic systems or any system that is characterized by growth habits for which the faces are crystallographically identical. However, that is not the case, because all crystallographic directions are represented nonetheless, even if only at rounded corners. Recent results on growth of hematite by OA show that, at a single value of the pH, crystals with very different growth habits all attach along the <001> direction *even when there is no (001) facet*[12].

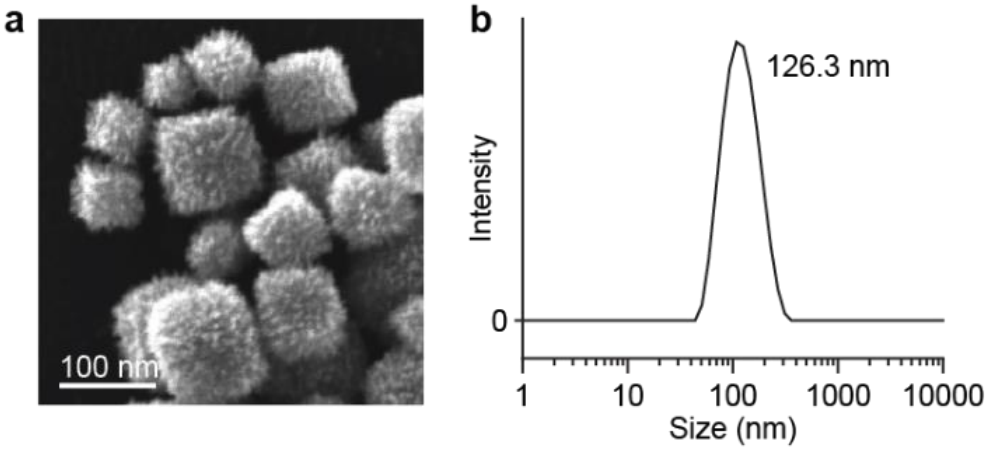

Supplementary Fig. 1: Synthesized branched cubic Pt mesocrystals. a,**b**, SEM image (**a**) and dynamic light scattering results (**b**) of synthesized branched cubic Pt mesocrystals. The average hydrodynamic diameter is 126.3 nm. Source data are provided as a Source Data file.

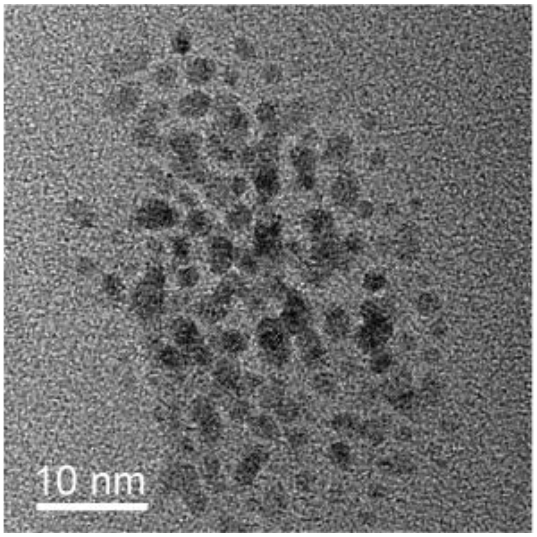

Supplementary Fig. 2: Evolution of Pt NPs at 15 min. Ex situ TEM image of Pt NPs observed at 15 min.

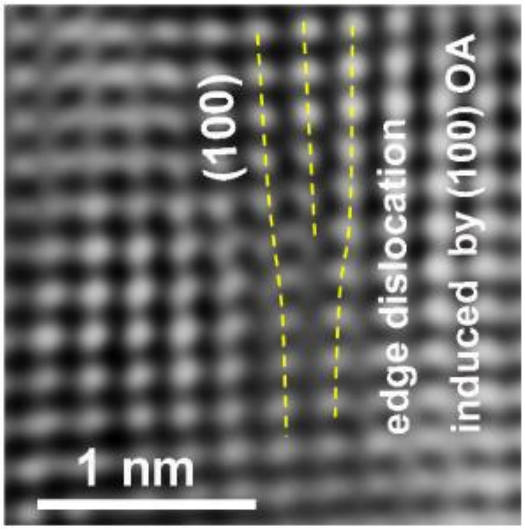

Supplementary Fig. 3: Edge dislocation in a Pt mesocrystal. Atomic-resolution STEM image of the branched cubic Pt mesocrystals showing the edge dislocation along {100} planes in the cubic core.

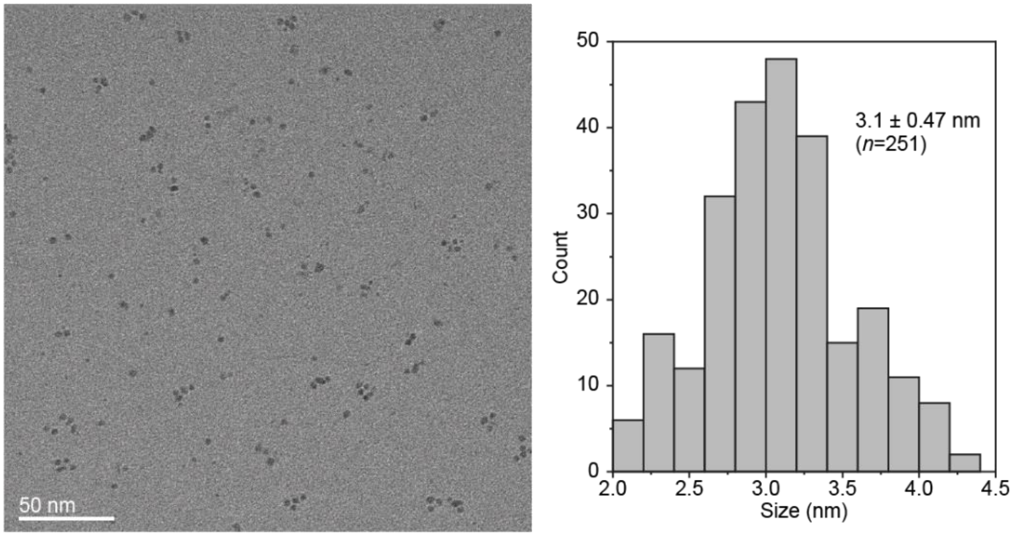

Supplementary Fig. 4: Characterization of primary NPs. LPTEM image and size distribution of primary NPs. Source data are provided as a Source Data file.

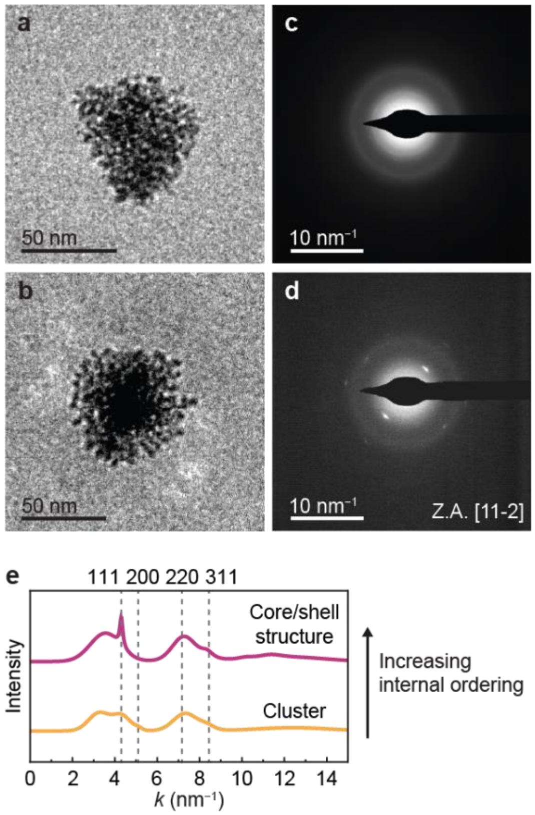

Supplementary Fig. 5: Structural analysis of cluster and core/shell structure. a–d, LPTEM images (**a**,**b**) and corresponding SAED patterns (**c**,**d**) of cluster (**a**,**c**) and core/shell structure (**b**,**d**). **e**, Radial distribution profiles from the SAED patterns in **c** and **d**, indicating the structural ordering of core/shell structure is higher than cluster. Source data are provided as a Source Data file.

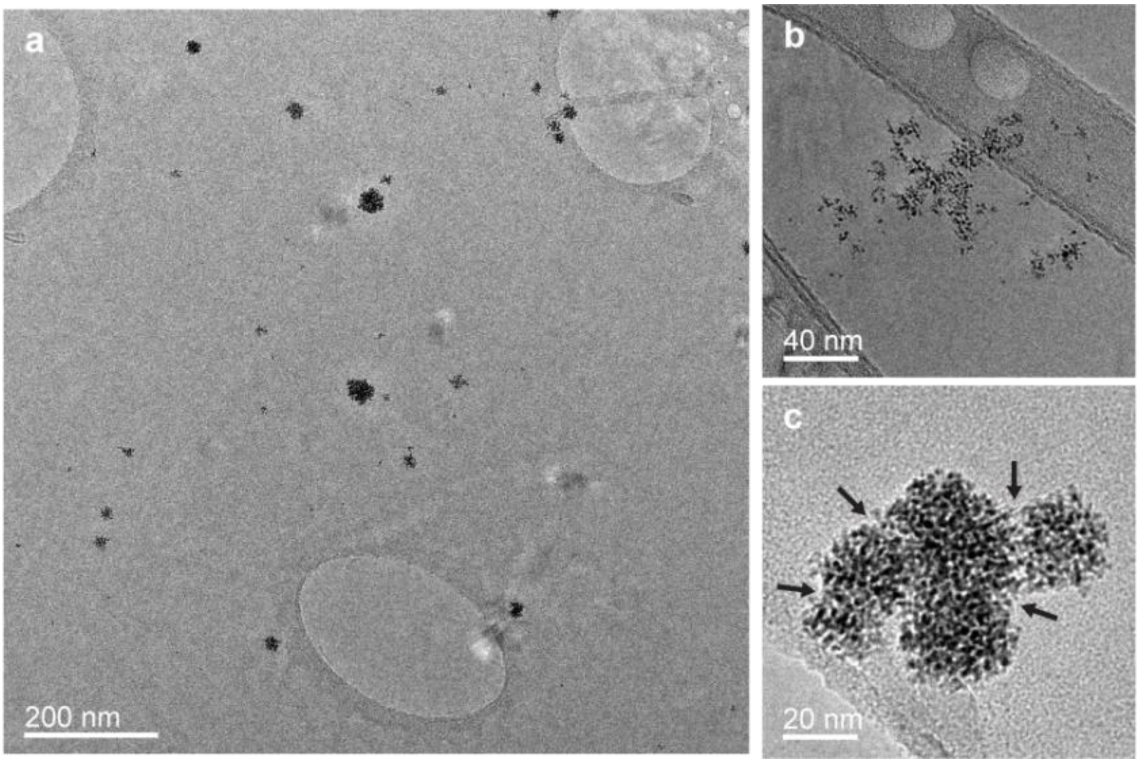

Supplementary Fig. 6: Cluster growth by aggregation. a−**c**, Cryo-TEM images showing clusters (**a**) and their growth by aggregation between NPs (**b**) and clusters (**c**). The concave neck between clusters in **c** is clear evidence of cluster growth by their aggregation.

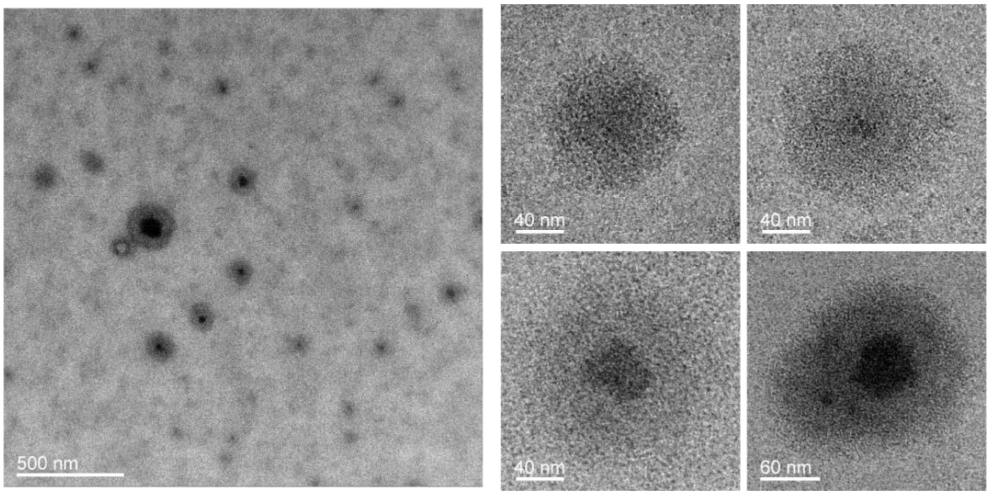

Supplementary Fig. 7: Intermediate structures in mesocrystal growth. LPTEM snapshot showing an intermediate stage in the growth solution of branched cubic Pt mesocrystals (left). Cloud-like clusters and core/shell-like structures (right).

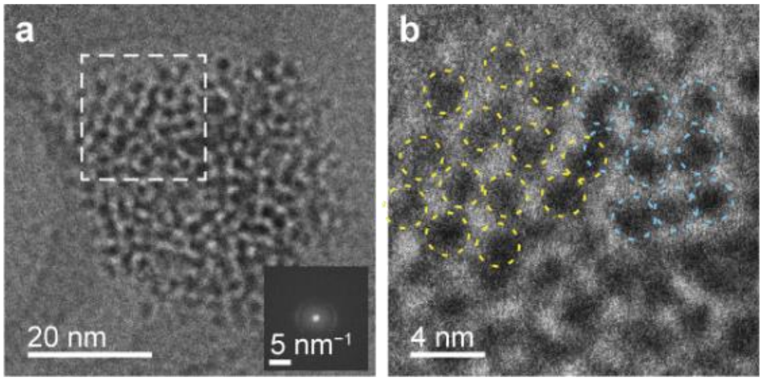

Supplementary Fig. 8: Ordered alignment of NPs in a cluster. a, HR-cryo-TEM image observed at 13 min of a cluster consisting of NPs with spacing. Inset, corresponding FFT patterns of **a**. **b**, The magnified image in **a** showing NPs aligned in a square-like pattern, marked by dashed circles.

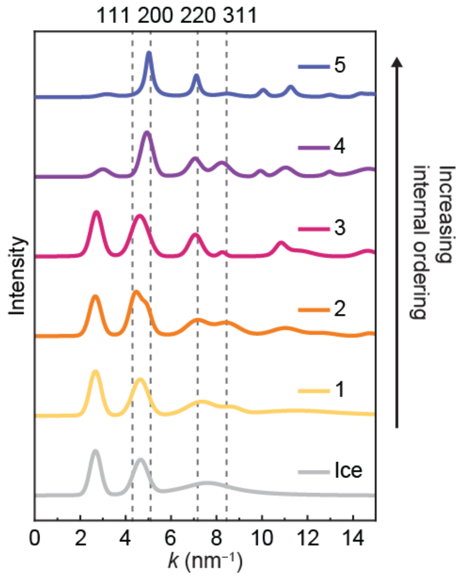

Supplementary Fig. 9: Evolution of structural order. Radial distribution profiles from the SAED patterns in Fig. 3f–j indicating the structural ordering gradually increases in the order of 1 < 2 < 3 < 4 < 5. Gray plot is from the SAED pattern of vitreous ice. 1-f; 2-g; 3-h; 4-i; and 5-j, respectively. Source data are provided as a Source Data file.

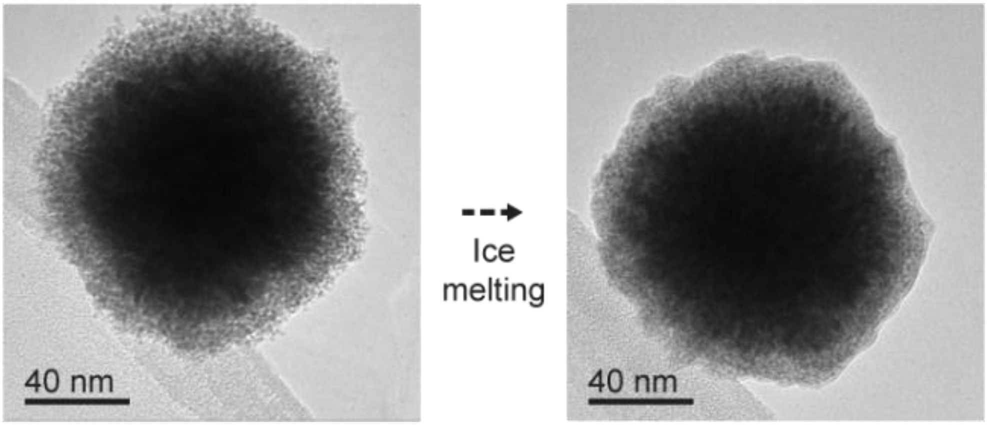

Supplementary Fig. 10: Beam-induced damage from solvent loss. Cryo-TEM images showing that the damage to the shell structure due to loss of solvent between the NPs when the surrounding ice layer is melted by the electron beam.

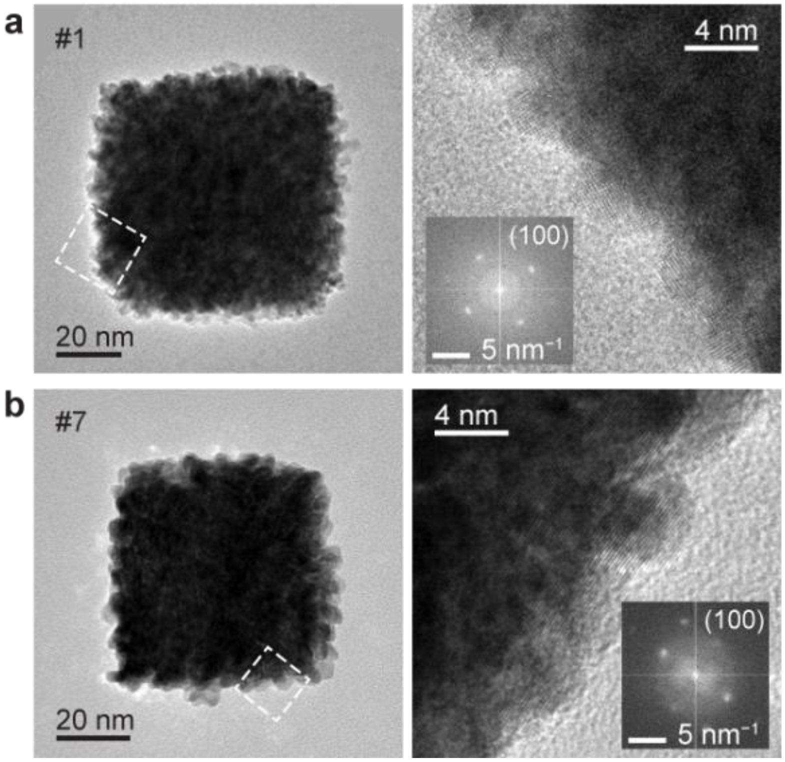

Supplementary Fig. 11: Synthesized cubic Pt mesocrystals. a,**b**, HR-TEM image observed 2 days after the reaction showing the resulting structure in which the OA of NPs does not switch to {111} attachment on the cube surface under low ratio of formic acid to K_2PtCl_4. **a**, condition #1; **b**, condition #7 in Supplementary Table 1, respectively. The initial concentration of K_2PtCl_4 and formic acid is 1.45 mM and 5.2 mM (**a**), and 4.8 mM and 52 mM (**b**), respectively.

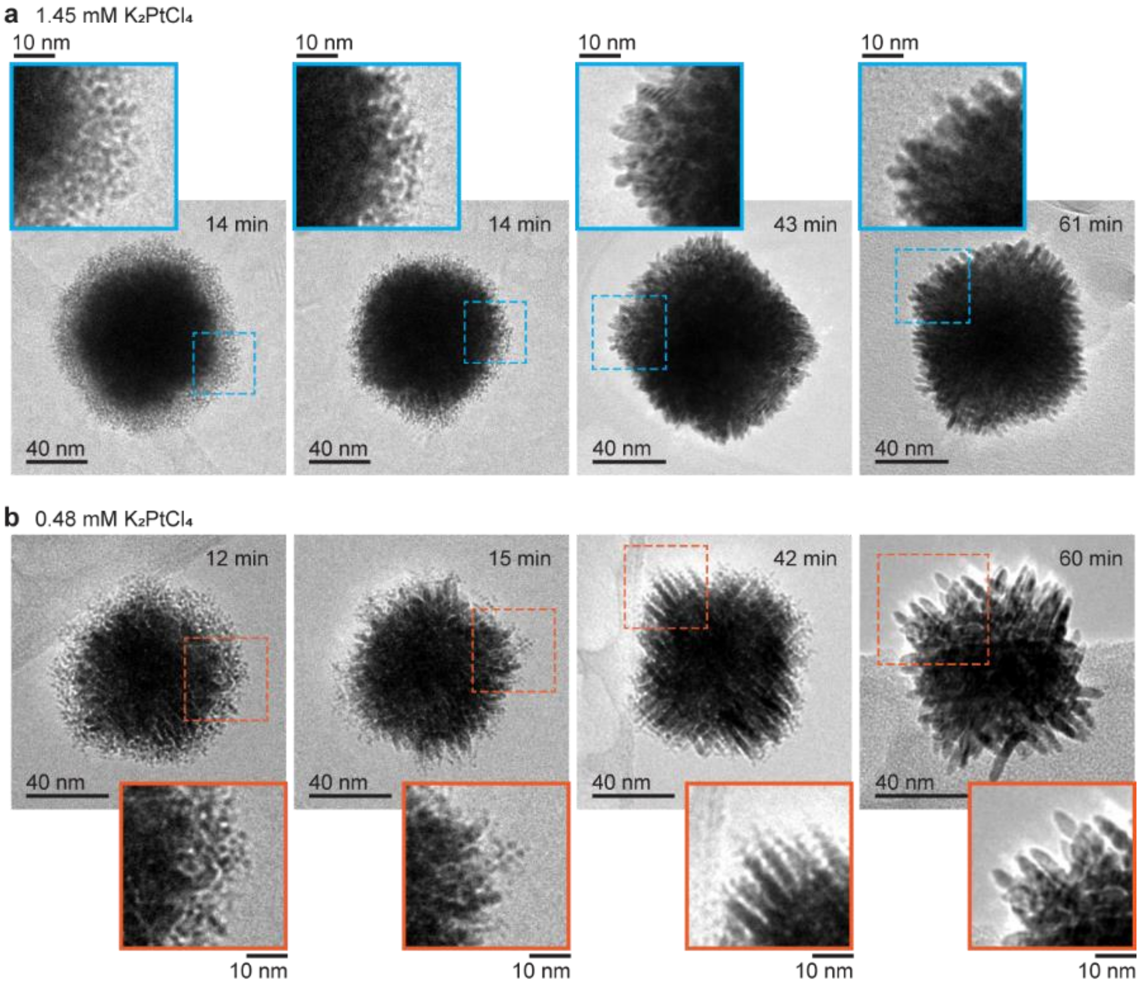

Supplementary Fig. 12: Effect of formic acid to Pt ratio on branch formation. a,**b**, Time-resolved cryo-TEM images at different initial concentration of K_2PtCl_4. Enlarged images show the emergence of branch rod. The initial concentration of K_2PtCl_4 is 1.45 mM (**a**) and 0.48 mM (**b**), respectively. The initial concentration of formic acid is 52 mM. **a**, condition #2; **b**, condition #6 in Supplementary Table 1, respectively.

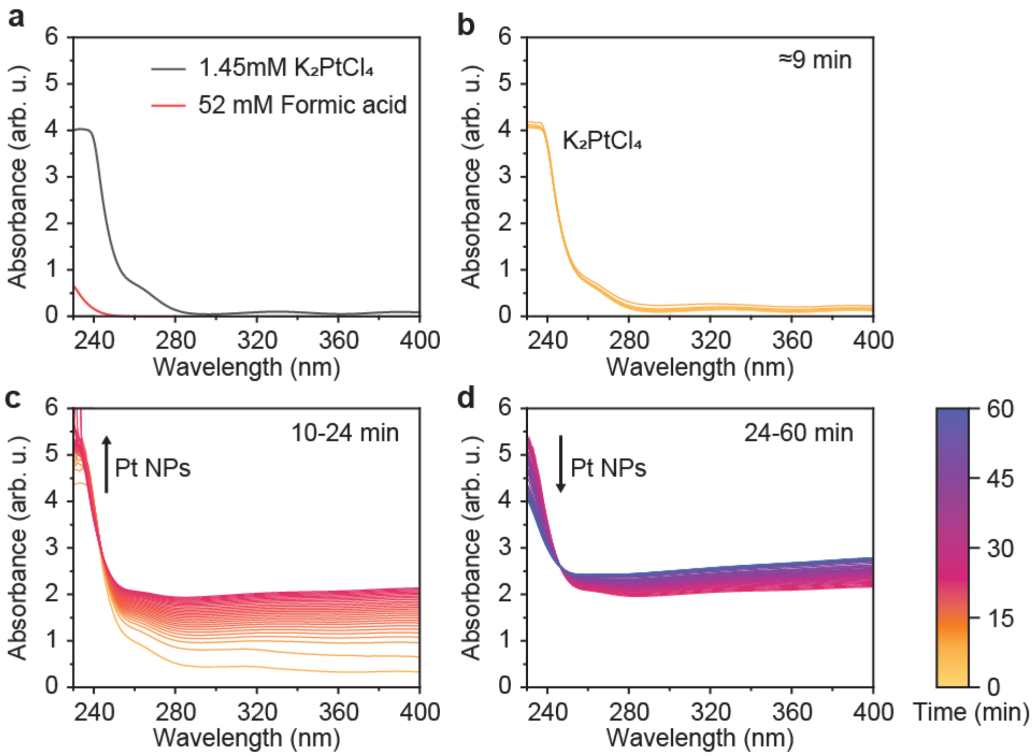

Supplementary Fig. 13: UV-vis analysis during mesocrystal growth. **a**, UV-vis absorbance spectra of 1.45 mM K_2PtCl_4 and 52 mM formic acid. **b**–**d**, In situ UV-vis absorbance spectra during the growth of branched cubic Pt mesocrystals with initial concentrations of K_2PtCl_4 and formic acid of 1.45 mM and 52 mM, respectively. K_2PtCl_4 shows until 9 min (**b**), the absorbance band of Pt NPs appears after 10 min and increases in intensity (**c**), and then decreases in intensity after 24 min (**d**). Source data are provided as a Source Data file.

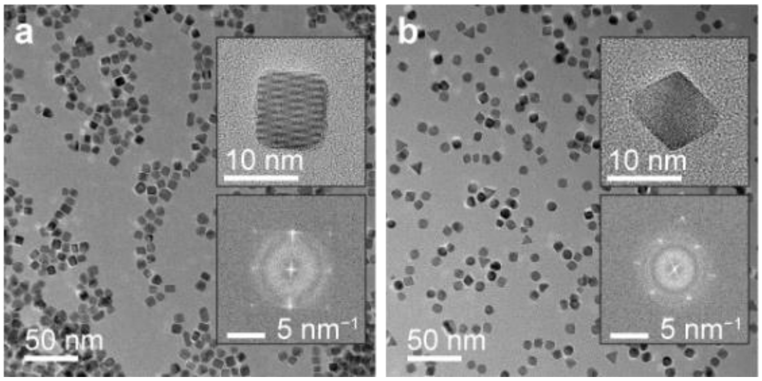

Supplementary Fig. 14: Pt NPs with {100} and {111} facets. a,**b**, TEM images and FFT patterns of ≈10 nm sized cube- (**a**) and octahedral-shaped Pt NPs (**b**) representing {100} and {111} facets, respectively.

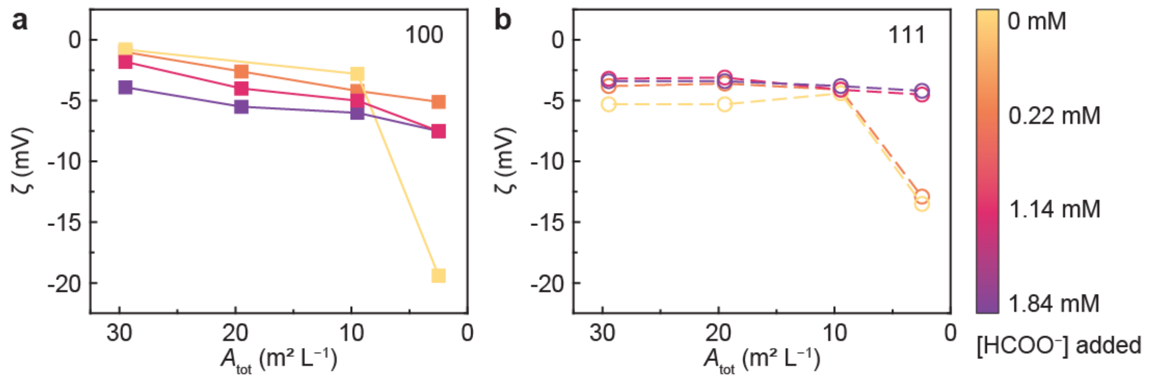

Supplementary Fig. 15: Facet-dependent zeta potential analysis. a,**b**, Zeta potential (ζ) of Pt{100} (**a**) versus Pt{111} (**b**) as a function of A_{tot} in solution containing 2.9 mM HCl, 2.9 mM KCl and 0 mM (yellow), 4 mM (orange), 26.5 mM (pink), and 50 mM (purple) HCOOH; [$HCOO^-$] in solution are 0 mM (yellow), 0.22 mM (orange), 1.14 mM (pink), and 1.84 mM (purple). Source data are provided as a Source Data file.

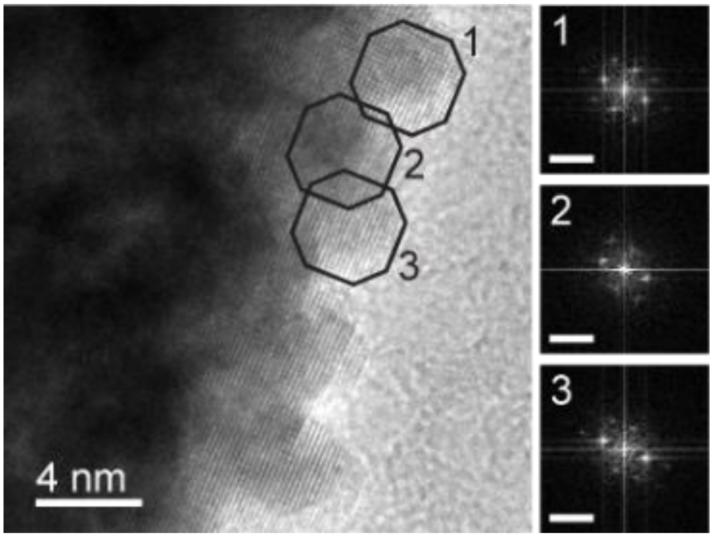

Supplementary Fig. 16: Shape of primary NPs. HR-TEM image and FFT patterns of Pt mesocrystals (condition #1 in Supplementary Table 1) observed 2 days after the reaction showing the truncated octahedral NPs attached with coherent orientation. Three FFT patterns are acquired from each NPs having the same numbering in the HR-TEM image. Scale bars, 10 nm^{-1}.

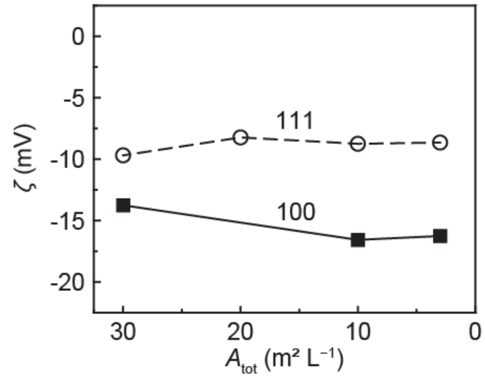

Supplementary Fig. 17: Facet-dependent zeta potential analysis. Zeta potential (ζ) of Pt{100} versus Pt{111} as a function of A_{tot} in solution containing 13.3 mM HCOOH ([$HCOO^-$] = [H^+] = 1.46 mM). Source data are provided as a Source Data file.

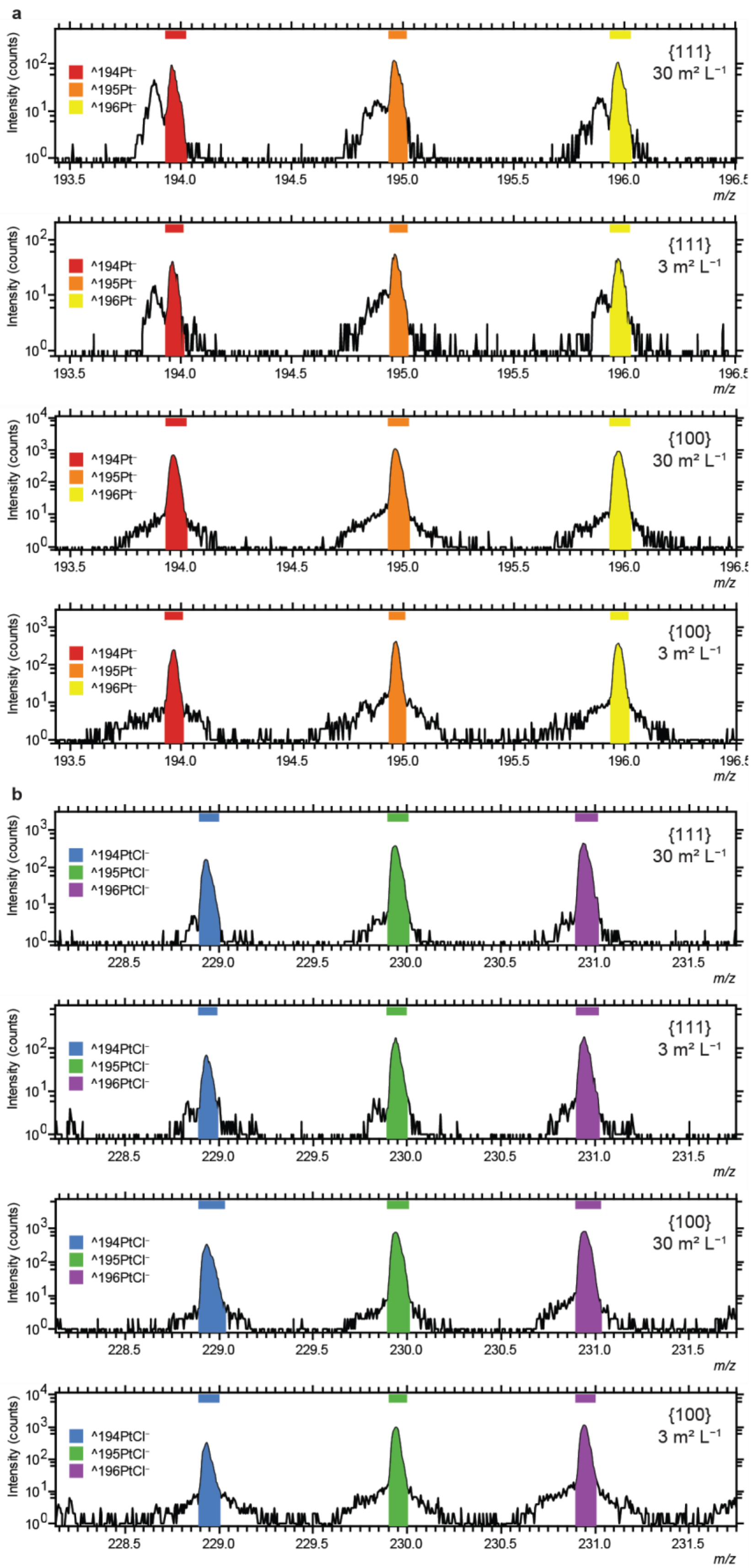
a
Intensity (counts)
^194Pt-
^195Pt-
^196Pt-
{111}
30 m² L⁻¹
3 m² L⁻¹
{100}
30 m² L⁻¹
3 m² L⁻¹
193.5
194.0
194.5
195.0
195.5
196.0
m/z
b
^194PtCl-
^195PtCl-
^196PtCl-
228.5
229.0
229.5
230.0
230.5
231.0
231.5
m/z

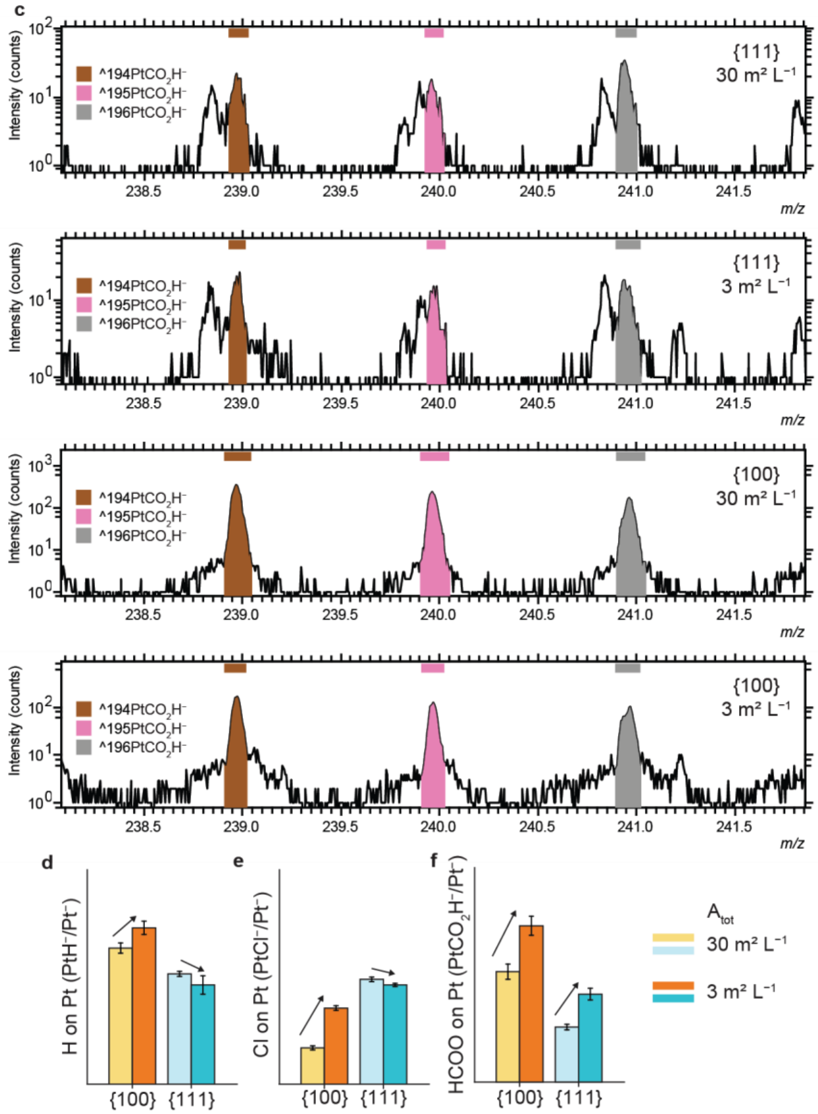

Supplementary Fig. 18: TOF-SIMS analysis of ion adsorption on Pt facets. a–c, TOF-SIMS spectra in the *m*/*z* ranges relevant for Pt^- (**a**), $PtCl^-$ (**b**), and $PtHCOO^-$ (**c**) ions of Pt NPs with predominantly {100} and {111} surfaces at A_{tot} of 30 m^2 L^{-1} and 3 m^2 L^{-1} in solution, with concentrations of H^+, K^+, Cl^- and $HCOO^-$ at 4.0 mM, 2.9 mM, 5.8 mM and 1.14 mM, respectively. **d–f**, The signal intensity of adsorbed H (**d**), Cl (**e**), and HCOO (**f**) on Pt {100} and {111} surfaces is normalized to that of Pt. Yellow, the {100} surface at A_{tot} of 30 m^2 L^{-1}; Orange, the {100} surface at A_{tot} of 3 m^2 L^{-1}; Light blue, the {111} surface at A_{tot} of 30 m^2 L^{-1}; Blue, the {111} surface at A_{tot} of 3 m^2 L^{-1}. Data points represent the mean of four independent measurements for the {100} surface at A_{tot} of 30 m^2 L^{-1} and the mean of five independent measurements for all other conditions. Error bars represent the standard deviation of four independent measurements for the {100} surface at A_{tot} of 30 m^2 L^{-1} and of five independent measurements for all other conditions. The small error bars reflect consistent measurements. Source data are provided as a Source Data file.

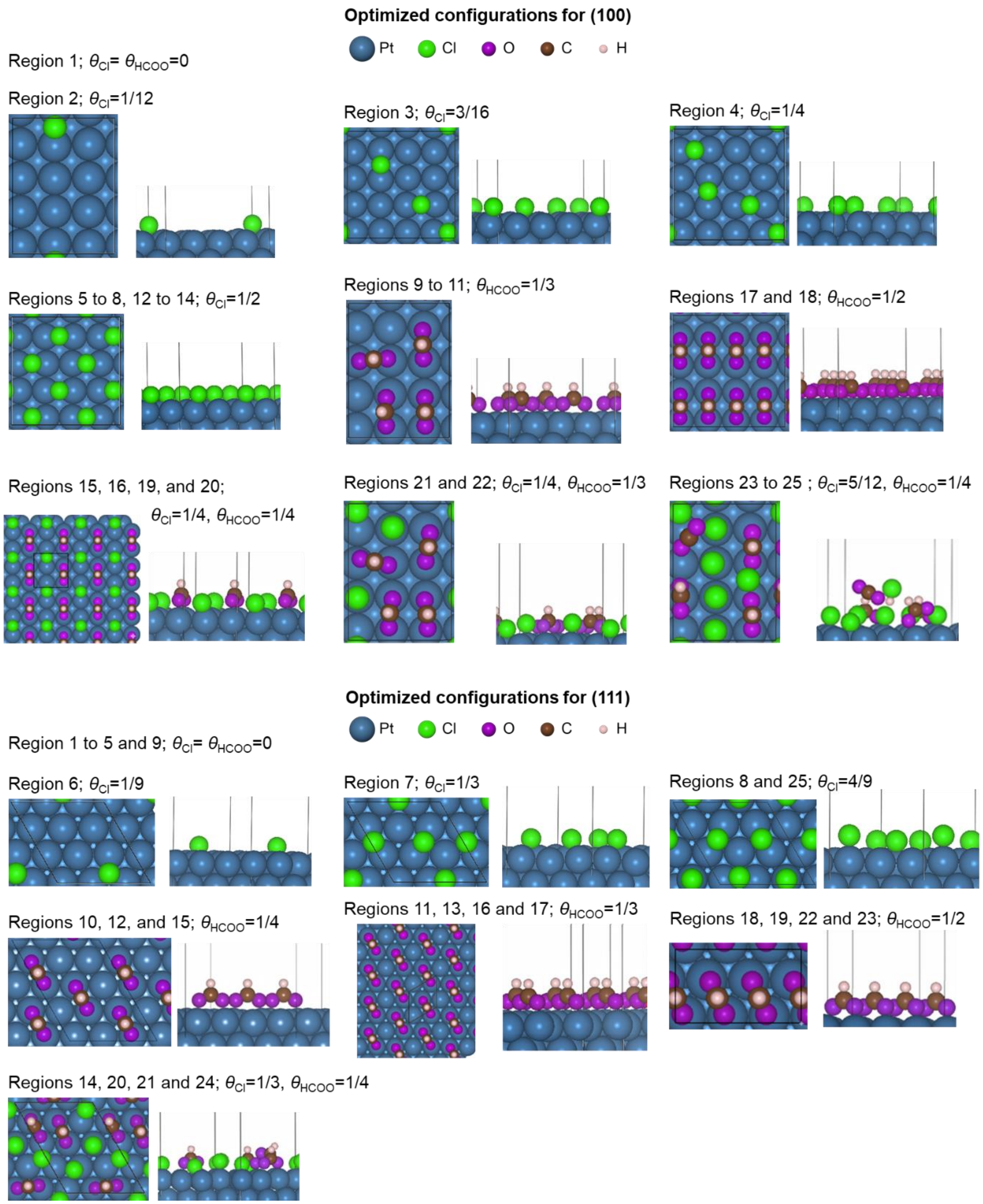

Supplementary Fig. 19: DFT-optimized surface configurations. Optimized final configurations of Pt(100) and Pt(111) surfaces using DFT. Blue, platinum; Green, chlorine; Purple, oxygen; Pink, hydrogen; Brown, carbon. θ is the coverage of each species, where θ=1 means there is one species for each Pt surface atom.

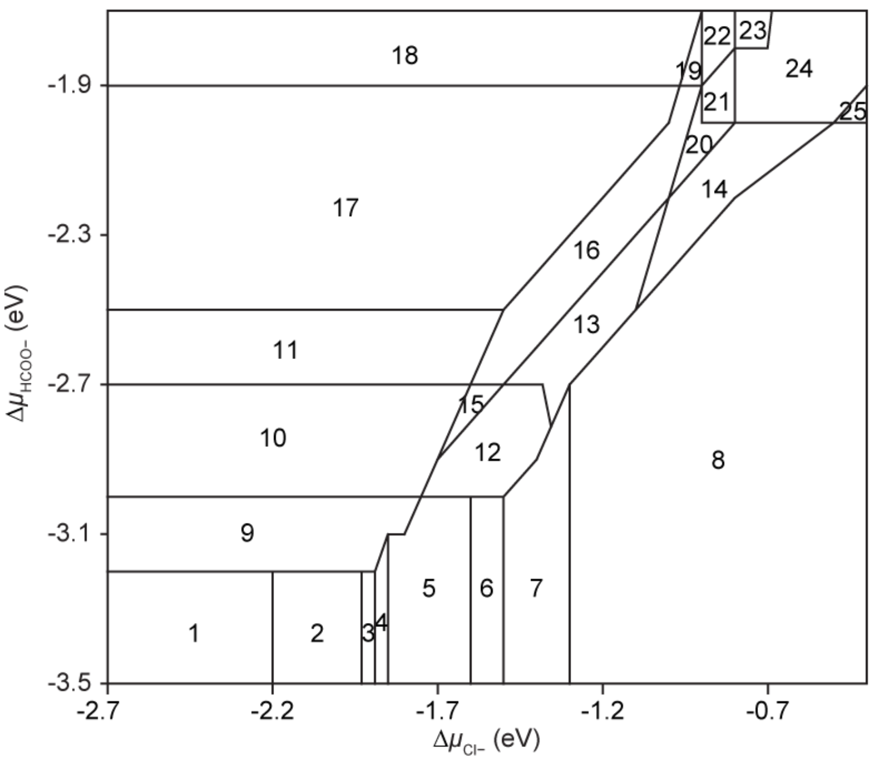

Supplementary Fig. 20: Surface energy diagram of Pt surfaces. Minimum surface-energy diagram as a function of the chemical potentials of Cl^{-} and $HCOO^{-}$ relative to the gas phase ($\Delta\mu_{Cl^-}$ and $\Delta\mu_{HCOO^-}$) on Pt(100) and Pt(111) surfaces, as calculated by DFT. θ and binding energies for each numbered region are given in Supplementary Table 2.

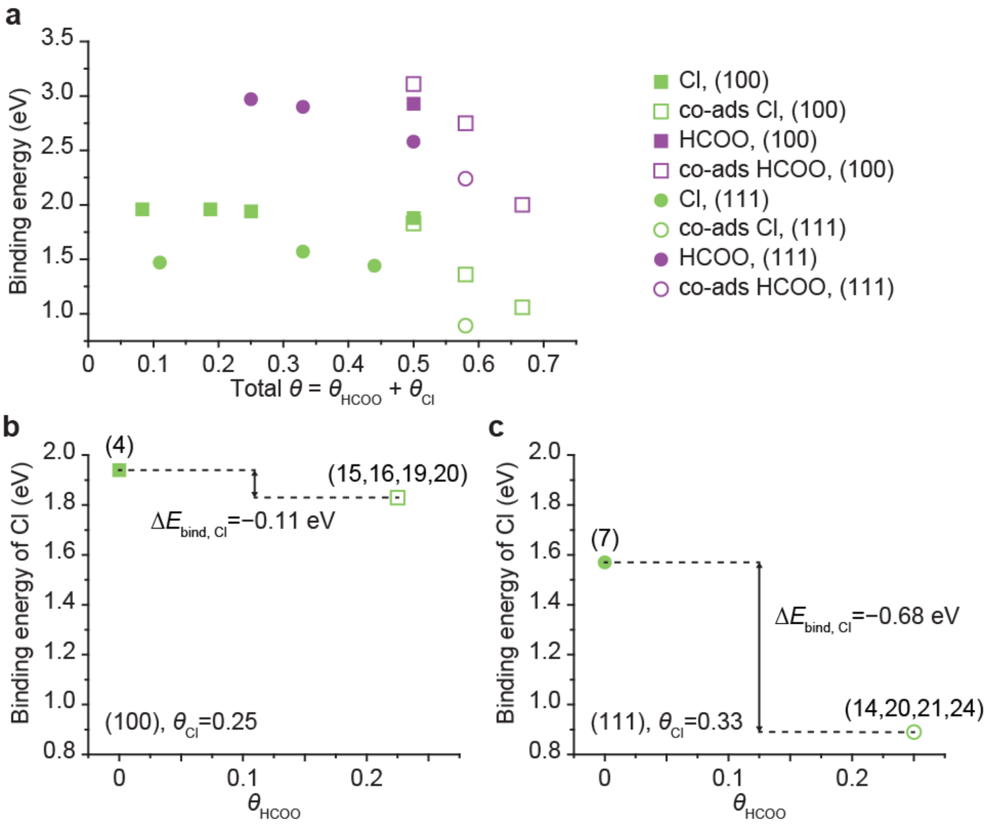

Supplementary Fig. 21: Binding energy analysis for co-adsorption on Pt surfaces. a, Binding energies (E_{bind}) of Cl and HCOO versus total coverage (θ) on Pt(100) and Pt(111). **b**, E_{bind} of Cl (at θ_{Cl}=0.25) as a function of θ_{HCOO} co-adsorbed on Pt(100). **c**, E_{bind} of Cl (at θ_{Cl}=0.33) as a function of θ_{HCOO} co-adsorbed on Pt(111). **b** and **c** show that as θ_{HCOO} increases, E_{bind} of Cl decreases more on Pt(111) than on Pt(100). Squares and circles represent the {100} and {111} facets, respectively; Cl is colored green and HCOO is purple. Colored symbols represent surfaces adsorbed by a single species, while open symbols represent co-adsorbed surfaces. The numbers in parentheses correspond to regions in Supplementary Fig. 20, and θ and E_{bind} for each numbered region are given in Supplementary Table 2, as calculated by DFT. θ is the coverage of each species, where θ=1 means there is one species for each Pt surface atom. Source data are provided as a Source Data file.

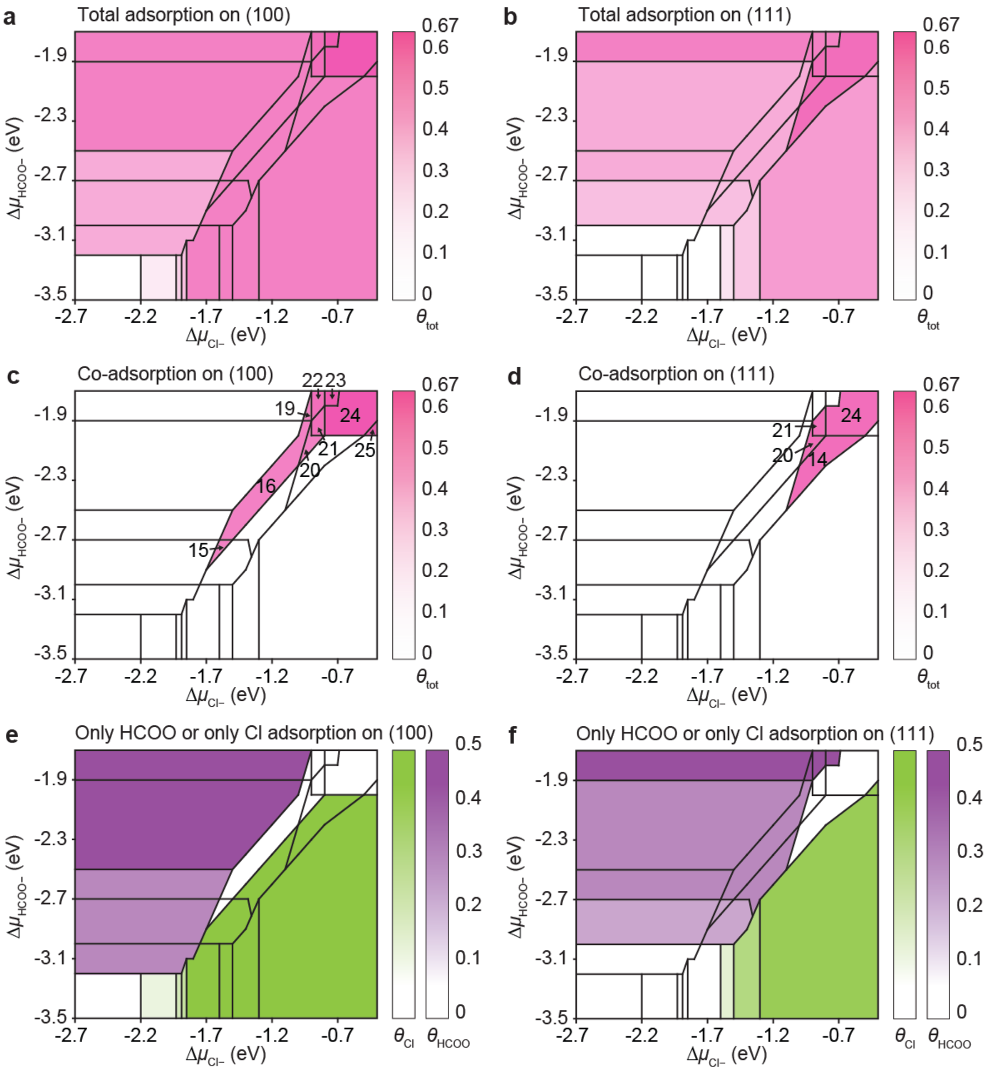

Supplementary Fig. 22: Detailed surface energy diagrams of Pt surfaces. a–f, Minimum surface-energy diagram as a function of the chemical potentials of Cl^- and $HCOO^-$ relative to the gas phase ($\Delta\mu_{Cl^-}$ and $\Delta\mu_{HCOO^-}$) on Pt(100) and Pt(111), as calculated by DFT. θ_{tot}, θ_{Cl}, and θ_{HCOO} represent the total coverage, the coverage of Cl, the coverage HCOO, respectively, where θ=1 means there is one species for each Pt surface atom. The colored regions indicate the different θ shown in the color bar on the right. θ_{tot} on (100) (**a**); θ_{tot} on (111) (**b**); θ_{tot} on co-adsorbed (111) (**c**); θ_{tot} on co-adsorbed (111) (**d**); θ_{HCOO} (purple) and θ_{Cl} (green) on either only HCOO adsorbed- or only Cl adsorbed (100) (**e**); and θ_{HCOO} (purple) and θ_{Cl} (green) on either only HCOO adsorbed- or only Cl adsorbed (111) (**f**). Regions of the full surface-energy diagram are shown in Supplementary Fig. 20. θ and binding energies for each numbered region are given in Supplementary Table 2.

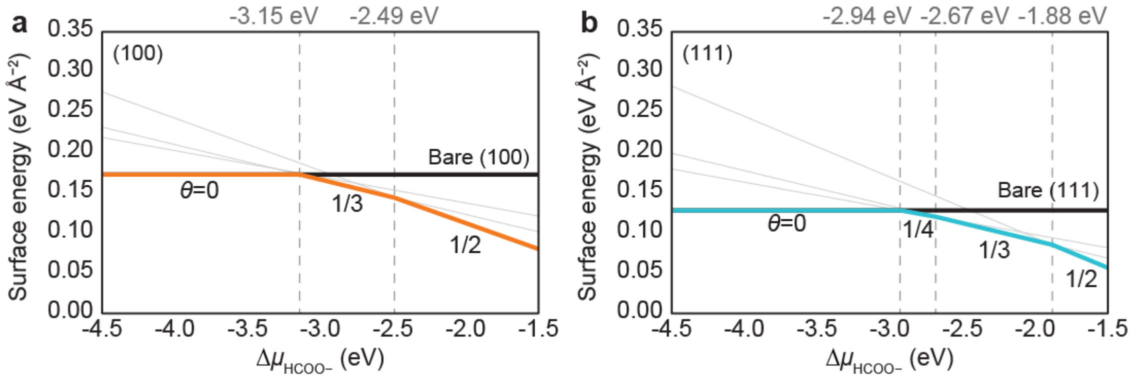

Supplementary Fig. 23: Surface energy diagram of formate adsorption. a,**b**, Surface phase diagram of Pt as a function of the chemical potential of $HCOO^-$ relative to the gas phase ($\Delta\mu_{HCOO^-}$) on the Pt(100) (**a**) and Pt(111) (**b**), respectively, as calculated by DFT. Gray line represents each surface calculated using equation (4). Black lines correspond to bare Pt(100) and Pt(111). Orange and blue lines show the lowest surface energy of (100) and (111) surfaces, respectively. θ is the coverage of HCOO on Pt surfaces with the lowest surface energy, where θ=1 means there is one species for each Pt surface atom. Gray top numbers and dashed vertical lines indicate the chemical potential values at which different HCOO coverages are favored. Binding energies in each region are indicated in Supplementary Table 2. Similar diagrams for Cl adsorption were published in ref. [13].

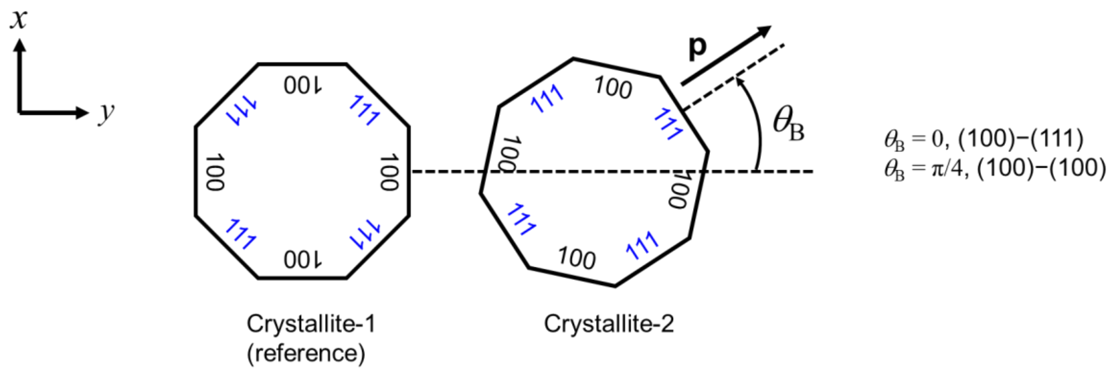

Supplementary Fig. 24: Schematic of geometric configuration for relative crystallite rotation. The case where the (100) of crystallite-1 faces crystallite-2 in the *xy* plane and crystallite-2 rotates with respect to one another. **p** is a unit vector perpendicular to (111) surface in this case.

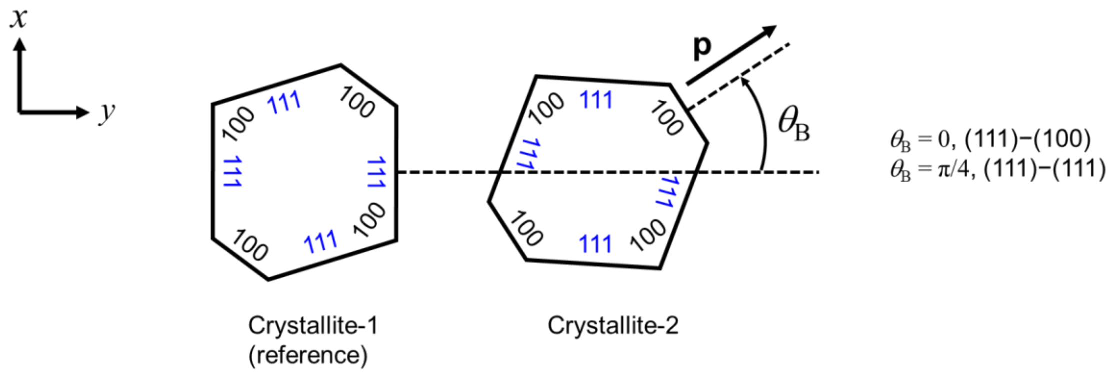

Supplementary Fig. 25: Schematic of geometric configuration for relative crystallite rotation. The case where the (111) of crystallite-1 faces crystallite-2 in the *xy* plane and crystallite-2 rotates with respect to one another. **p** is a unit vector perpendicular to (100) surface in this case.

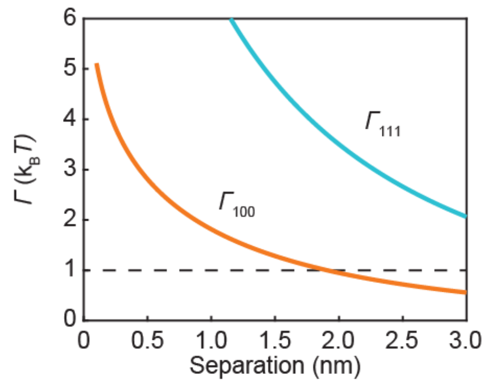

Supplementary Fig. 26: Calculated torque for specific NP alignment. Torque required to align NPs along (100)–(100) (Γ_{100}) and (111)–(111) (Γ_{111}) as a function of separation distance between two NPs.

Supplementary Table 1

Synthesis conditions numbered in Fig. 4 and Supplementary Figs. 11, 12 and the calculated concentration of each species after assuming complete reduction of K_2PtCl_4 and structural features of the final product. After reducing K_2PtCl_4, the remaining HCOOH partially dissociates into $HCOO^-$ and H^+, with their concentrations determined by the equilibrium constant and the stabilized pH. Units are mM.

	Before reduction			After reduction								
#	K_2PtCl_4	HCOOH	Initial pH	K^+	Cl^-	HCOOH	$HCOO^-$	H^+	Final pH	$HCOO^-$/Pt	Cube	Branch
1	1.45	5.2	3.06	2.9	5.8	3.54	0.21	3.11	2.51	0.14	O	Little
2	1.45	52	2.53	2.9	5.8	48.70	1.85	4.75	2.32	1.27	O	Short
3	1.45	520	2.02	2.9	5.8	510.3	8.24	11.14	1.95	5.68	O	Long
4	0.15	5.2	3.05	0.3	0.6	4.31	0.74	1.04	2.98	4.97	O	Long
5	0.48	5.2	3.05	0.96	1.44	4.20	0.52	1.48	2.83	1.07	O	Short
6	0.48	52	2.53	0.96	1.44	48.99	2.53	3.49	2.46	5.27	O	Long
7	4.8	52	2.53	9.6	14.4	46.40	0.80	10.40	1.98	0.17	O	Little

Supplementary Table 2

Coverage (θ) and binding energy (E_{bind}) of Cl and HCOO on Pt(100) and Pt(111) for each numbered region in Fig. 6e,f and Supplementary Figs. 20−23, from DFT calculations. θ=1 means there is one species for each Pt surface atom.

	Pt(100)				Pt(111)			
	θ		E_{bind} (eV)		θ		E_{bind} (eV)	
Region	Cl	HCOO	Cl	HCOO	Cl	HCOO	Cl	HCOO
1	0	0	-	-	0	0	-	-
2	0.083	0	1.93	-	0	0	-	-
3	0.1875	0	1.96	-	0	0	-	-
4	0.25	0	1.94	-	0	0	-	-
5	0.5	0	1.88	-	0	0	-	-
6	0.5	0	1.88	-	0.11	0	1.47	-
7	0.5	0	1.88	-	0.33	0	1.57	-
8	0.5	0	1.88	-	0.44	0	1.44	-
9	0	0.33	-	3.16	0	0	-	-
10	0	0.33	-	3.16	0	0.25	-	2.97
11	0	0.33	-	3.16	0	0.33	-	2.9
12	0.5	0	1.88	-	0	0.25	-	2.97
13	0.5	0	1.88	-	0	0.33		2.9
14	0.5	0	1.88	-	0.33	0.25	0.89	2.24
15	0.25	0.25	1.83	3.11	0	0.25	-	2.97
16	0.25	0.25	1.83	3.11	0	0.33	-	2.9
17	0	0.5	-	2.93	0	0.33	-	2.9
18	0	0.5	-	2.93	0	0.5	-	2.58
19	0.25	0.25	1.83	3.11	0	0.5	-	2.58
20	0.25	0.25	1.83	3.11	0.33	0.25	0.89	2.24
21	0.25	0.33	1.36	2.75	0.33	0.25	0.89	2.24
22	0.25	0.33	1.36	2.75	0	0.5	-	2.58
23	0.417	0.25	1.06	2.00	0	0.5	-	2.58
24	0.417	0.25	1.06	2.00	0.33	0.25	0.89	2.24
25	0.417	0.25	1.06	2.00	0.44	0	1.44	-

Supplementary Table 3

Unit cell and its corresponding k mesh are used for DFT geometry optimization. Centered unit cells are used, noted by *c*.

	Pt(100)			Pt(111)		
Unit cell	*c* (2×3)	*c* (3×4)	*c* (4×4)	*c* (3×3)	*c* (3×4)	*c* (4×4)
k points	(7×5×1)	(9×6×1)	(5×5×1)	(7×7×1)	(8×8×1)	(8×6×1)

Supplementary references

1. Doi, M. & Edwards, S. F. *The Theory of Polymer Dynamics*. (Clarendon Press, Oxford, 2013).
2. Ohshima, H. Effective surface potential and double-layer interaction of colloidal particles. *J. Colloid Interface Sci.* **174**, 45–52 (1995).
3. Wijenayaka, L. A., Ivanov, M. R., Cheatum, C. M. & Haes, A. J. Improved parametrization for extended Derjaguin, Landau, Verwey, and Overbeek predictions of functionalized gold nanosphere stability. *J. Phys. Chem. C* **119**, 10064–10075 (2015).
4. Liu, L. *et al.* Connecting energetics to dynamics in particle growth by oriented attachment using real-time observations. *Nat. Commun.* **11**, 1045 (2020).
5. Lee, J. *et al.* Effects of particle shape and surface roughness on van der Waals interactions and coupling to dynamics in nanocrystals. *J. Colloid Interface Sci.* **652**, 1974–1983 (2023).
6. Lee, J. *et al.* Defect self-elimination in nanocube superlattices through the interplay of Brownian, van der Waals, and ligand-based forces and torques. *ACS Nano* **18**, 32386–32400 (2024).
7. Lifshitz, E. M. & Hamermesh, M. The theory of molecular attractive forces between solids. in *Perspectives in Theoretical Physics* (ed. Pitaevski, L. P.) 329–349 (Pergamon, Amsterdam, 1992). doi:10.1016/B978-0-08-036364-6.50031-4.
8. Parsegian, V. A. & Weiss, G. H. Dielectric anisotropy and the van der Waals interaction between bulk media. *J. Adhes.* **3**, 259–267 (1972).
9. Hamaker, H. C. The London—van der Waals attraction between spherical particles. *Physica* **4**, 1058–1072 (1937).

10. Chatman, S., Zarzycki, P. & Rosso, K. M. Surface potentials of (001), (012), (113) hematite (α-Fe2O3) crystal faces in aqueous solution. *Phys. Chem. Chem. Phys.* **15**, 13911 (2013).

11. Su, S. *et al.* Facet-dependent surface charge and hydration of semiconducting nanoparticles at variable pH. *Adv. Mater.* **33**, 2106229 (2021).

12. Wang, Y. *et al.* Particle-based hematite crystallization is invariant to initial particle morphology. *Proc. Natl. Acad. Sci. U.S.A.* **119**, e2112679119 (2022).

13. Kim, E. M., Kim, J. & Fichthorn, K. A. A study of Cl adsorption on Pt(111) and Pt(100) using ab initio grand-canonical Monte Carlo. *Surf. Sci.* **752**, 122647 (2025).
