# Supplementary material for: Anisotropic surface potentials induced by competitive ion adsorption enable the synthesis of branched cubic Pt mesocrystals
Source: Nat Commun. 2025 Nov 5;16:9758. doi: 10.1038/s41467-025-64494-9 (PMC12589459; doi:10.1038/s41467-025-64494-9)
Supplement: Supplementary file 2 — Description of Additional Supplementary Files [file 41467_2025_64494_MOESM2_ESM.pdf]

Description of Additional Supplementary Files

File Name: Supplementary Movie 1

Description: In situ LPTEM movie showing early-stage cluster growth by NP aggregation. The movie plays in real time. Dimension is 70 nm by 70 nm.

File Name: Supplementary Movie 2

Description: In situ LPTEM movie showing further cluster growth by their aggregation. The movie plays in real time. Dimension is 100 nm by 100 nm.

File Name: Supplementary Movie 3

Description: In situ LPTEM movie showing rearrangement of NPs in square-like pattern within a cluster. The movie plays in real time. Dimension is 70 nm by 70 nm.
